# Neonatal Health Following IVF: Own Versus Donor Material in Singleton and Multiple Pregnancies

**DOI:** 10.3390/life15040578

**Published:** 2025-04-01

**Authors:** Lucia Elena Niculae, Raluca Tocariu, Evelyn-Denise Archir, Alexandru-Ștefan Niculae, Anca-Magdalena Coricovac, Diana-Elena Comandașu, Aida Petca, Elvira Brătilă

**Affiliations:** 1 Department of Obstetrics and Gynecology, “Carol Davila” University of Medicine and Pharmacy, 8 Eroii Sanitari Blvd., 050474 Bucharest, Romania; lucia-elena.ghirca@drd.umfcd.ro (L.E.N.); diana.comandasu@drd.umfcd.ro (D.-E.C.); aida.petca@umfcd.ro (A.P.); elvirabarbulea@gmail.com (E.B.); 2 Department of Neonatology, Clinical Hospital of Obstetrics and Gynecology “Prof. Dr. Panait Sârbu”, 3–5 Giulesti St, 060251 Bucharest, Romania; evelyn-denise.archir@rez.umfcd.ro; 3 Department of Pediatric Neurology, “Carol Davila” University of Medicine and Pharmacy, 8 Eroii Sanitari Blvd., 050474 Bucharest, Romania; alexandru-stefan.niculae@rez.umfcd.ro; 4 Department of Embryology, Faculty of Dentistry, “Carol Davila” University of Medicine and Pharmacy, 8 Eroii Sanitari Blvd., 050474 Bucharest, Romania; anca.coricovac@umfcd.ro; 5 Gynera Fertility Center, 020308 Bucharest, Romania; 6 Department of Obstetrics and Gynecology, Clinical Hospital of Obstetrics and Gynecology “Prof. Dr. Panait Sârbu”, 3–5 Giulesti St, 060251 Bucharest, Romania; 7 Department of Obstetrics and Gynecology, Elias University Emergency Hospital, 17 Mărăști Blvd., 050474 Bucharest, Romania

**Keywords:** in vitro fertilization, donor gametes, neonatal outcomes, assisted reproductive technology

## Abstract

This study investigates neonatal outcomes in singleton and multiple pregnancies following in vitro fertilization (IVF) using donor (IVF-D) versus autologous (IVF-A) material. A retrospective cohort analysis was conducted with 988 neonates born between 2017 and 2024 across three tertiary neonatal units in Romania. The primary outcomes included preterm birth, low birthweight, neonatal asphyxia, and congenital malformations. IVF-D pregnancies were associated with a higher prevalence of adverse neonatal outcomes, particularly in multiple gestations. Preterm birth and low birthweight were more frequent in the IVF-D group, with donor-conceived neonates exhibiting increased rates of neonatal ventilation and prolonged hospitalization. Additionally, congenital anomalies, particularly cardiac malformations, were more prevalent in IVF-D pregnancies, suggesting possible immunological and epigenetic influences. Despite these differences, overall neonatal survival was comparable between groups. These findings contribute to the existing literature on assisted reproductive technologies, emphasizing the need for further research to clarify the biological mechanisms influencing neonatal outcomes and to optimize the clinical management of IVF pregnancies using donor gametes.

## 1. Introduction

Since the first successful oocyte donation in 1984 [1], in vitro fertilization (IVF) with donor material has become a vital option for women facing infertility due to diminished ovarian reserves, genetic disorders, advanced maternal age, or iatrogenic causes such as cancer treatments [2]. Nowadays, due to a social phenomenon favoring delayed childbearing in modern societies, assisted reproductive technology (ART) programs relying on gamete donation are permitted in the majority of countries worldwide [3,4].

Despite its growing medical necessity, research has consistently identified an elevated risk of adverse obstetric and perinatal outcomes, such as preterm birth and low birthweight, when compared to spontaneous pregnancies and IVF with autologous oocytes and sperm [5,6,7]. Although the precise biological mechanisms underlying these associations remain uncertain, maternal factors, particularly advanced age, may play a significant role. In the general population, advanced maternal age, often a characteristic of donor oocyte recipients, is a well-documented risk factor for complications including preeclampsia, gestational diabetes, preterm labor, and cesarean delivery. These age-related risks complicate efforts to determine whether adverse outcomes are primarily driven by the use of donor material or the demographic profile of the recipients [8,9].

Prompted by the observations mentioned above, the objective of our study was to investigate whether medically indicated IVF with gamete donation (IVF-D) is associated with poor neonatal outcomes in singleton and multiple pregnancies compared to IVF with autologous material (IVF-A).

## 2. Materials and Methods

### 2.1. Study Sample and Data Collection

This observational, retrospective cohort study consisted of singleton and multiple live births after donor or autologous material IVF cycles, following ESHRE guidelines, from three third-degree neonatal units based in Bucharest and Constanța, Romania. Following approval from each individual Ethics Committee, maternal data pertaining to ART procedures were sourced from the Clinical Hospital of Obstetrics and Gynecology “Prof. Dr. Panait Sârbu” Bucharest and Gynera Fertility Center, whereas detailed information regarding maternal health parameters and neonatal outcomes was systematically obtained from the three neonatal units where the deliveries occurred. Data were collected from 1 January 2017 to 1 June 2024 through the electronic systems of each healthcare facility, with strict measures in place to ensure the confidentiality and security of the information.

Women were included if they had a donor or autologous IVF cycle, followed by a singleton or multiple live birth (>22 weeks’ gestation). The primary indications for donor oocyte IVF in our cohort included advanced maternal age, diminished ovarian reserve, premature ovarian failure, and genetic conditions incompatible with autologous conception. Baseline maternal and neonatal characteristics including maternal age, a status of pregnancy-induced arterial hypertension and gestational diabetes mellitus, gestational age at delivery, birthweight, and Apgar score at 1 and 5 min were collected. The estimation of gestational age at delivery was based on the date of the embryo transfer. Clinical characteristics, such as the mode of fertilization (IVF vs. ICSI), cycle (natural vs. artificial), type of embryo (fresh vs. frozen), day of embryo transfer, type of material (donor vs. own) and cause of infertility, were included. We chose to exclude women who underwent preimplantational genetic testing or those with incomplete records.

### 2.2. Outcome Measures

The primary outcomes assessed were preterm birth (PTB), neonatal asphyxia, low birthweight (LBW), and macrosomia. PTB was defined as delivery before 37 weeks of gestation. Birth asphyxia was noted if the Apgar score at 1 min was lower than 7, based on the newborn’s inability to initiate efficient breaths. LBW was defined as a birthweight of less than 2500 g, reflecting restricted fetal growth or preterm delivery, while macrosomia was characterized by a birthweight exceeding 4000 g, often linked to maternal diabetes or prolonged gestation. Secondary outcomes included the following: congenital malformations, the duration of invasive and non-invasive ventilation (if required), the length of hospital stays, and neonatal death occurring during the first 28 days of life. Neonates were hospitalized primarily due to prematurity-related complications (respiratory distress syndrome and the need for ventilation), congenital anomalies, neonatal asphyxia, and low birthweight requiring specialized neonatal care. Each primary and secondary outcome was evaluated independently for single and multiple births to distinctly capture variations associated with different pregnancy types.

### 2.3. Statistical Analysis

Data analysis was conducted using JASP 0.19.2.0. Categorical variables were analyzed using the Chi-squared test and, for each primary outcome, odds ratios (ORs) were calculated to quantify the strength and direction of the association. Adjusted odds ratios (aOR) and confidence intervals (aCI) were not calculated due to the limited sample size in the donor group as a potential source of variability in the adjusted estimates. In all statistical analyses, including Chi-squared and Fisher’s exact tests, the alternative hypothesis assumed a higher incidence in the donor group compared to the autologous group. Continuous variables, including days of ventilation and hospitalization, were analyzed using the Mann–Whitney U test. Assumptions of normality and homogeneity of variance for the continuous variables were evaluated using the Shapiro–Wilk test and the Brown–Forsythe test, respectively. Additionally, Kendall’s Tau correlation coefficient was employed to assess the strength and direction of ordinal or non-linear relationships between variables, providing a robust measure of association for non-parametric data. Statistical significance was set to a *p*-value of <0.05 (two-tailed) in all analyses.

## 3. Results

Between January 2017 and June 2024, we systematically collected data on 988 neonates conceived via ART procedures using either donor or autologous material. This cohort included 670 neonates from singleton pregnancies and 318 neonates from multiple pregnancies, derived from 156 twin gestations and 2 triplet pregnancies. Maternal baseline characteristics are presented in Table 1 and Table 2. Kendall’s Tau correlations are attached as Supplementary Material.

Advanced maternal age (≥35 years) was significantly more common in IVF-D pregnancies within the singleton group (*p* < 0.001), while in multiples, this trend was notable but not statistically significant (*p* = 0.056). Pregnancy-induced hypertension occurred more frequently in IVF-D pregnancies for both groups, with an unadjusted odds ratio of 1.7 (95% CI 0.98–2.42) for singletons and 1.17 (95% CI 0.11–2.23) for multiples, though the latter had a marginally significant *p*-value of 0.032. Gestational diabetes showed a significantly higher occurrence in IVF-D pregnancies solely in the singleton group, with an OR of 0.86 (95% CI 0.1–1.63; *p* = 0.027), while multiples displayed no significant difference. Notably, some large odds ratios for IVF-D outcomes, even with significant *p*-values, may reflect reduced statistical precision due to the limited sample size of the donor group.

Given the distinct physiological and clinical differences between these groups, separate analyses were conducted for singletons and multiples to ensure a more nuanced and comprehensive evaluation of neonatal outcomes associated with IVF.

### 3.1. Singletons Conceived Through IVF Donor vs. Autologous Material

In singleton pregnancies, neonatal outcomes demonstrated notable differences between those conceived via IVF using donor and autologous material, revealing clinically relevant patterns (see Table 3). The median birthweight was marginally lower in the donor group (3130 g, IQR 590, range 920–4000) than the comparison group (3190 g, IQR 640, range 590–4680), potentially reflecting subtle discrepancies in fetal growth trajectories but with no statistical significance. Gestational age was comparable, with a median of 38 weeks in both groups, suggesting similar timing of delivery irrespective of the type of IVF material used.

Apgar scores at 1 min were consistent between groups, both presenting a median of 9. However, at 5 min, the IVF-D group exhibited a lower median score (7 vs. 8), and the proportion of neonates with an Apgar score ≤ 7 was significantly higher in the IVF-D cohort (*p* = 0.02; OR 3.8, 95% CI 1.36–10.57). This finding may indicate a diminished neonatal adaptation at birth in IVF-D pregnancies, warranting further investigation and postnatal monitoring.

Preterm birth (<37 weeks) was more prevalent in the IVF-D group, with an OR of 2.09 (95% CI 1.02–4.29; *p* = 0.038). Although rates of low birthweight (<2500 g), congenital malformations, and neonatal death were higher following gamete donation, these differences were not statistically significant. Furthermore, the wide confidence intervals for some outcomes, such as neonatal death (OR 5.83, 95% CI 0.59–57.48), underscore the need for larger cohorts and the importance of cautious interpretation.

Birthweight percentiles were assigned using the Fenton growth chart to assess neonatal growth in singleton pregnancies conceived via in vitro fertilization and each neonate was classified as small for gestational age (SGA under 10th percentile), appropriate for gestational age (AGA between 10th and 90th percentile), and large for gestational age (LGA over 90th percentile). A chi-square test was conducted to compare the distribution of SGA versus non-SGA neonates conceived with donor and autologous material. The analysis yielded no statistically significant difference between the two groups (χ^2^ = 0.042, *p* = 0.838), supported by the odds ratio of 0.81 (*p* = 0.719), suggesting that fetal growth trajectories in singleton pregnancies were comparable, regardless of gamete origin. The contingency table, with the odds ratio analysis, can be found in the Supplementary Material.

**Figure 1:**
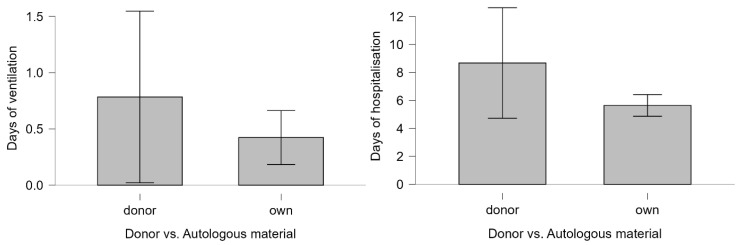
Bar plots with singletons conceived through IVF donor vs. autologous material and days of ventilation/hospitalization.

The requirement for neonatal ventilation was markedly more frequent in the donor group, with an unadjusted OR of 4.34 (95% CI 1.67–11.29; *p* = 0.007). Similarly, extended hospitalization exceeding three days had an OR of 2.44 in IVF-D singletons (95% CI 1.25–4.75; *p* = 0.008). These findings align with the Mann–Whitney U test results, which further revealed longer durations of ventilation (U = 13,082.000, *p* < 0.001) and hospital stays (U = 14783.000, *p* < 0.001) in the IVF-D group (Figure 1, Supplementary Material). Neonates conceived following IVF with donor material had higher mean ventilation days (0.784 vs. 0.423 in IVF-A) and length of hospitalization (8.676 vs. 5.641 days), suggesting a heightened risk of postnatal complications, with prolonged medical support.

In the analysis of neonatal outcomes by sex among singleton pregnancies, no statistically significant differences were observed. Female and male neonates had comparable gestational ages (median 38 weeks), birthweights (3150 g vs. 3200 g), Apgar scores at 1 min, and lengths of hospital stays. Mann–Whitney U tests indicated no significant sex-related disparities across these parameters (all *p*-values > 0.1), suggesting similar neonatal adaptation in both groups (see Supplementary Material for full statistical analysis).

### 3.2. Multiples Conceived Through IVF Donor vs. Autologous Material

Consistently, in multiple pregnancies, neonatal outcomes were significantly less favorable for those conceived via IVF-D compared to IVF-A, with donor-conceived neonates experiencing disproportionately higher rates of adverse perinatal outcomes (see Table 4).

Birthweight was notably lower in the donor group, with a median of 2030 g compared to 2360 g in the autologous group, while gestational age at delivery followed a similar pattern, with a median of 34 weeks versus 36 weeks, respectively. The increased prevalence of preterm birth in the IVF-D group (90.9% vs. 68.0%, *p* < 0.001) highlights the compounded vulnerability of donor-conceived multiples to premature delivery, suggesting that oocyte or sperm donation may further exacerbate the inherent risks associated with multiple gestation. However, while the odds of being small for one’s gestational age appeared higher in the donor gamete group (OR = 1.776), this association did not achieve statistical significance (χ^2^ = 1.739, *p* = 0.187; *p* = 0.143 for the odds ratio analysis), indicating that any observed differences in multiples are likely attributable to chance, rather than a meaningful biological effect (Supplementary Material).

Neonatal adaptation was also less favorable in the IVF-D group, as reflected in significantly lower Apgar scores at 1 min, where the incidence of birth asphyxia was 63.6% for donor-conceived neonates compared to 33.8% in the IVF-A group (*p* < 0.001). Although Apgar scores at 5 min did not differ significantly between groups, the trend toward lower values in the donor group may indicate a more prolonged transition to extrauterine life.

**Figure 2:**
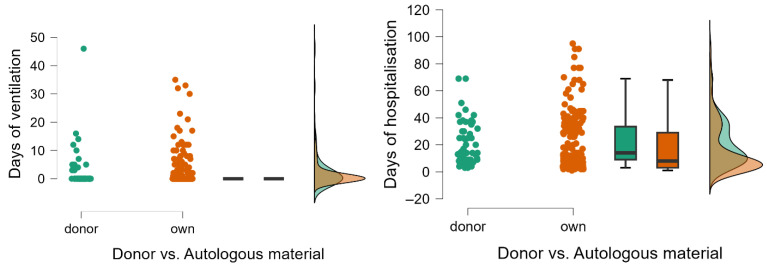
Raincloud plots with multiples conceived through IVF donor vs. autologous material and days of ventilation/hospital stay.

Prolonged ventilation was more frequent in the donor group (23.6% vs. 15.6%), though this difference did not reach statistical significance (*p* = 0.1). Similarly, Mann–Whitney U analysis showed that mean ventilation days were slightly higher in the IVF-D group (2.436 days, SD = 7.018) compared to the IVF-A group (1.962 days, SD = 5.338), but with a *p*-value of 0.469, suggesting that while donor-conceived multiples may experience more respiratory complications, the variability within groups limits definitive conclusions (Figure 2, Supplementary Material).

In contrast, the duration of hospitalization was significantly longer for donor-conceived multiples, reinforcing findings observed in singleton pregnancies. The median hospital stay was notably increased in the IVF-D group (21.418 days, SD = 16.120) compared to the IVF-A group (17.152 days, SD = 19.787), with Mann–Whitney U analysis confirming statistical significance (U = 9468.000, *p* < 0.001). Nearly all IVF-D multiples (96.4%) required hospitalization beyond three days, compared to 71.9% in the IVF-A group (*p* < 0.001). This trend mirrors findings in singletons, where prolonged hospitalization was also significantly associated with donor conception (*p* < 0.001).

Furthermore, congenital malformations were significantly more frequent in IVF-D multiples (27.3% vs. 15.6%, *p* = 0.034), suggesting a possible association between gamete donation and an increased risk of structural anomalies. Although the exact mechanisms remain unclear, potential explanations include epigenetic modifications associated with donor material or variations in embryonic development [10,11].

Meanwhile, neonatal mortality rates remained comparable between groups (1.8% vs. 1.9%, *p* = 0.68), though the small number of cases limits the ability to draw definitive conclusions.

It is important to acknowledge that some adverse neonatal outcomes observed in both groups are primarily attributable to multiple gestation itself, independent of donated material. Multiple pregnancies inherently carry an increased risk of prematurity, low birthweight, and neonatal morbidity due to factors such as uteroplacental insufficiency, heightened fetal demands, and a greater likelihood of obstetric interventions [12,13]. The high prevalence of preterm birth in the IVF-A group (68%) reflects this baseline risk, suggesting that some of the observed differences between IVF-D and IVF-A groups may be a result of the additive effects of multiple gestation and donor conception, rather than donor status alone. Nonetheless, the significantly poor outcomes in the donor group, particularly in terms of gestational age, birthweight, and hospital stay, suggest that gamete donation may introduce additional biological factors that warrant further investigation.

Among multiple pregnancies, neonatal outcomes were likewise comparable between sexes. Gestational age, birthweight, Apgar scores, and hospitalization metrics did not differ significantly between male and female neonates. Non-parametric testing confirmed the absence of statistically significant sex-based differences (all *p*-values > 0.1), indicating that neonatal adaptation following multiple gestations was not influenced by sex in this cohort (see Supplementary Material).

### 3.3. Prevalence and Distribution of Congenital Malformations (Singletons vs. Multiples)

**Figure 3:**
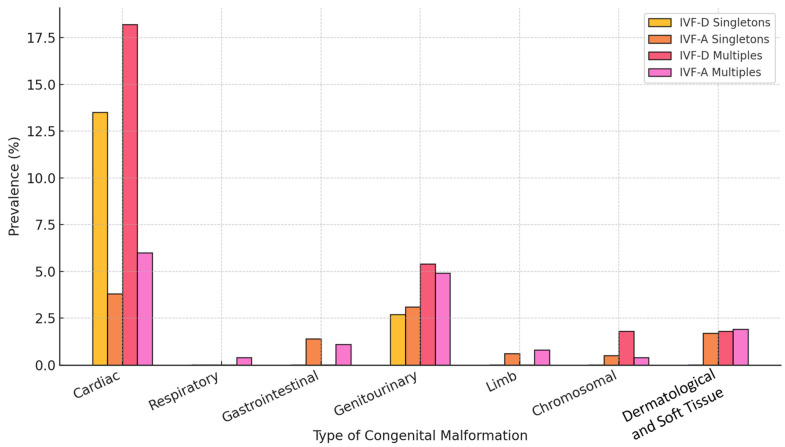
Distribution of congenital malformations in singletons and multiples (IVF-D vs. IVF-A).

As mentioned, the prevalence of congenital malformations was higher in neonates conceived via IVF with donor material compared to those conceived with autologous material in both singleton and multiple pregnancies, with a more pronounced difference observed in multiples. Among singletons, congenital anomalies were identified in 16.2% of IVF-D neonates compared to 11.2% in the IVF-A group. This disparity was even greater in multiple pregnancies, where 27.3% of IVF-D neonates had congenital malformations compared to 15.6% in the IVF-A group (Figure 3, Supplementary Material).

Cardiac anomalies were the most frequently reported malformations in both groups, with their prevalence significantly higher in IVF-D pregnancies. In singletons, 13.5% of donor-conceived neonates had a cardiac defect compared to 3.8% in the autologous group, while in multiples, the difference was similarly striking (18.2% vs. 6%). Patent foramen ovale, atrial septal defects, and ventricular septal defects were among the most frequently observed conditions, with additional cases of coarctation of the aorta and pulmonary artery stenosis appearing in multiples. The increased frequency of cardiac malformations in donor-conceived neonates aligns with prior research suggesting an association between ART and congenital heart defects, although the underlying mechanisms are not yet fully understood [14].

Malformations affecting other organ systems were also more prevalent in IVF-D groups, though the differences were less pronounced. Gastrointestinal anomalies, including ankyloglossia and cleft lip/palate, were rare but present in both groups, while genitourinary abnormalities, such as hydronephrosis and cryptorchidism, were slightly more frequent in donor-conceived neonates. Limb malformations, chromosomal, or genetic disorders, and other miscellaneous anomalies were observed at lower frequencies, but the overall trend suggested an increased risk in IVF-D conceptions.

These findings should be interpreted with caution due to the limited sample size of the donor group, particularly in singletons (n = 37), which may affect statistical power and result in variability. Larger studies are needed to validate these associations and clarify potential underlying mechanisms.

## 4. Discussion

### 4.1. Key Findings

Our study identified notable differences in neonatal outcomes between IVF pregnancies using donor and autologous material, with donor-conceived neonates exhibiting higher rates of adverse perinatal events. In singleton pregnancies, IVF-D was associated with a higher incidence of preterm birth, lower birthweight, and reduced Apgar scores, suggesting greater neonatal vulnerability at birth. These neonates demonstrated an increased need for respiratory support and had extended hospital stays compared to their IVF-A counterparts. Congenital malformations were also more prevalent, with cardiac anomalies being the most frequently observed. In multiple pregnancies, the burden of prematurity was even more pronounced in the IVF-D group, with nearly all neonates being delivered preterm and at lower birthweights. The need for prolonged hospitalization was significantly higher, and congenital anomalies, particularly cardiac defects, remained the most frequently reported. However, neonatal mortality was low in both groups, with no significant differences observed between IVF-D and IVF-A neonates.

Neonatal growth patterns, as assessed by birthweight percentiles, appeared comparable between singletons and multiples conceived with autologous and donor gametes. While some variation was observed, particularly in multiple pregnancies, no statistically significant differences emerged, suggesting that gamete origin does not meaningfully influence fetal growth trajectories.

In our cohort, the vast majority of pregnancies resulted in cesarean delivery, most of which were scheduled electively with limited or no trials of labor. This is consistent with established clinical practice for IVF-conceived pregnancies, which are typically managed as high-risk due to maternal comorbidities, advanced maternal age, and the use of donor material. Given the limited number of vaginal deliveries, a comparative statistical analysis of delivery modes was not feasible.

As for maternal comorbidities, significant differences between IVF-D and IVF-A pregnancies were observed, particularly regarding the prevalence of pregnancy-induced hypertension and gestational diabetes mellitus. Hypertensive disorders were significantly more frequent in donor-conceived pregnancies, particularly among singletons, whereas gestational diabetes showed a higher prevalence in IVF-D singletons.

### 4.2. Comparison with Existing Literature

These findings align with a growing body of evidence indicating that IVF with donor material is associated with an increased risk of adverse neonatal outcomes. Consistent with prior studies, our results demonstrate higher rates of preterm birth, low birthweight, and an increased need for neonatal respiratory support among donor-conceived neonates. Previous large-scale analyses, such as those by Malchau et al. and Kamath et al., have similarly reported a heightened risk of prematurity and small-for-gestational-age neonates following donor oocyte conception, with odds ratios comparable to those observed in our cohort [5,6]. Furthermore, our findings reinforce data from Boulet et al., who highlighted a significantly higher prevalence of low birthweight in singleton donor-conceived pregnancies, further suggesting that factors beyond maternal age, such as immunological and epigenetic influences, may play a role in neonatal health outcomes [7]. Additionally, our study identified a greater prevalence of congenital malformations, particularly cardiac anomalies, among donor-conceived neonates, mirroring prior research that has linked assisted reproductive technologies with an increased incidence of structural defects. The observed higher rates of prolonged hospitalization and neonatal ventilation in our study support findings from Schwartz et al., who documented increased rates of neonatal morbidity in donor-conceived pregnancies [9].

### 4.3. Explanation of Results

Pregnancies involving donor oocytes (also referred to in modern reproductive technology as “surrogate motherhood”) often exhibit a complex immune response at the maternal–fetal interface, as the fetus is allogenic to the mother [11]. This can create a host vs. graft rejection-like phenomenon, with significant immuno-inflammatory reactions, leading to placental dysfunction and thus contributing to adverse neonatal outcomes. For example, Kogan et al. found that placentas from IVF with donor eggs showed a high incidence of central ischemic infarctions, massive perivillous fibrin deposition, and an incomplete remodeling of spiral arteries, which can result in intrauterine growth restriction [15]. Furthermore, Gundogan et al. reported a significantly high number of placentas with severe chronic deciduitis and increased numbers of T helper and natural killer cells in the donor IVF group, which can be the basis for hypertensive disorders and preterm delivery [16]. This finding was further supported by Modest et al. after a large-scale analysis demonstrated that donor IVF pregnancies carry a higher risk for ischemic placental disease, defined as preeclampsia, placental abruption, and/or small-for-gestational-age neonates, in comparison to autologous and non-IVF pregnancies [17,18].

Building on the role of immuno-inflammatory dysregulation at the maternal–fetal interface, epigenetic alterations, and molecular disruptions may contribute to the increased risk of congenital malformations in IVF using donor material. DNA methylation, crucial for normal gene expression, is frequently disrupted in ART pregnancies, with Choux et al. demonstrating altered methylation patterns in IVF/ICSI-derived placentas. Similarly, histone modifications, which regulate chromatin structure and gene expression, show deviations in IVF-conceived embryos, potentially leading to abnormal development [19,20]. Beyond epigenetics, the dysregulation of the renin–angiotensin system, essential for cardiovascular formation, has been linked to congenital heart defects, with Wang et al. reporting an increased expression of AGTR1 and CTGF in IVF-conceived myocardial tissue [21]. This may explain our finding that cardiac anomalies were the most frequently reported malformations in IVF-D pregnancies, with a significantly higher prevalence compared to the autologous group in both singletons and multiples [22]. Beyond overt cardiac anomalies, emerging evidence suggests that subtle vascular disturbances, such as microvascular remodeling defects and endothelial dysregulation, may also originate from placental dysfunction, particularly in pregnancies conceived via donor gametes. Histopathological analyses of placentas from ART-conceived pregnancies have indicated impaired angiogenic signaling and aberrant vascular development, supporting the notion of a broader spectrum of placental vascular pathology [23]. Finally, while suboptimal culture conditions, including variations in oxygen tension and culture media, may exacerbate these risks, the use of donor material introduces additional genetic variability, with donor selection criteria unable to fully eliminate inherited susceptibilities [24,25,26].

The association between maternal comorbities, including hypertensive disorders and gestational diabetes mellitus, and poor neonatal outcomes has been consistently studied in the last decade. On the one hand, pregnancy-induced hypertension is associated with the vascular maladaptation of the uteroplacental unit, leading to placental hypoperfusion and ischemia. Therefore, chronic fetal hypoxia, intrauterine growth restriction, and an increased risk of preterm birth have been noted, reaching a peak in donor-conceived pregnancies possibly in response to allogeneic fetal antigens [27,28,29]. On the other hand, gestational diabetes mellitus exerts its effects primarily through maternal hyperglycemia, fetal hyperinsulinemia, and excessive fetal nutrient transfer, predisposing neonates to macrosomia, altered lung maturation, and neonatal metabolic instability. Chronic fetal exposure to maternal hyperglycemia results in pancreatic beta-cell hypertrophy in the fetus, leading to neonatal hypoglycemia following birth due to persistent hyperinsulinemia [30]. Additionally, hyperglycemia also disrupts surfactant synthesis and alveolar development, contributing to an increased risk of respiratory complications, even in late-preterm and term neonates [31]. Although this study did not perform an adjusted analysis to isolate the independent effects of these conditions, the higher prevalence of these comorbidities in the donor group suggests a multifactorial interplay between maternal health, immune tolerance, placental function, and fetal adaptation. Future research should aim to elucidate the specific contributions of these maternal risk factors to neonatal morbidity in IVF-conceived pregnancies, with a particular focus on immune-mediated mechanisms in donor oocyte pregnancies and the metabolic programming effects of gestational diabetes.

### 4.4. Strengths and Limitations

The main strength of this report lies in the large sample size collected from multiple tertiary neonatal units over a seven-year period, enhancing the generalizability of our findings. Also, our research provides a differentiated analysis by stratifying outcomes based on singleton and multiple gestations, hence offering a more precise understanding of how gamete donation impacts neonatal health across different pregnancy types while addressing a limitation in many previous reports. Another key strength of our study is the detailed assessment of congenital malformations and the incorporation of maternal risk factors, such as pregnancy-induced hypertension and gestational diabetes. By explicitly addressing these factors, our study adds a layer of clinical relevance, emphasizing the potential role of maternal health in mediating risks associated with donor conception. Lastly, our study employs a robust statistical methodology, including non-parametric tests such as the Mann–Whitney U test and Kendall’s Tau correlation, to account for the constraints of small sample sizes in the donor group.

However, there are certain limitations that need to be addressed, emphasizing the need for continued research in this area.

In this study, the calculation of adjusted odds ratios and confidence intervals was not feasible due to limitations within the dataset. A primary challenge was the imbalance in sample sizes, particularly the relatively small number of neonates in the donor-conceived group compared to the autologous group. This discrepancy resulted in wide and unstable confidence intervals, indicating high uncertainty in the estimates. Additionally, this issue was further exacerbated by the inherent variability in multiple gestations; neonatal outcomes are influenced by a complex interplay of factors, preventing meaningful adjustments. Furthermore, while we categorized pregnancies based on the use of donor or autologous material, we were unable to distinguish whether the donation involved oocytes, sperm, or both due to data collection constraints. This limitation prevents a more granular analysis of whether specific types of gamete donation may have varying effects on neonatal outcomes. Moreover, our dataset lacks information on maternal body mass index (BMI), a critical omission given the well-established association between obesity, infertility, and adverse perinatal events. The absence of BMI data constrains our ability to account for its potential confounding effects, particularly in the context of assisted reproductive technologies, where maternal metabolic health may be a key determinant of neonatal well-being.

Moreover, under the current legislative framework in Romania, gamete donation—whether involving oocytes or sperm—is not legally permissible. All donor material used in the included IVF procedures was imported from countries where donation is legally regulated and limited to anonymous donors. Consequently, the use of related or familial donors is not applicable within our clinical context. In addition, given that surrogacy is legally prohibited in Romania, our cohort did not include any surrogate pregnancies, precluding comparative analyses involving this subgroup.

Another key limitation of our study is the lack of an independent analysis evaluating the potential impact of the mode of delivery on neonatal respiratory outcomes. Given that nearly all IVF pregnancies in our cohort were delivered via cesarean section (CS), we were unable to perform a meaningful statistical comparison between CS and vaginal delivery. While prematurity remains the primary driver of neonatal respiratory distress syndrome in IVF-conceived neonates, it is important to acknowledge that elective CS without prior labor may also contribute to respiratory morbidity. The absence of labor-induced physiological adaptations, such as catecholamine-mediated lung fluid clearance, has been associated with increased rates of transient tachypnea of the newborn and respiratory distress syndrome [32,33,34].

Finally, chorionicity data were not available in all cases, limiting our ability to perform a separate analysis. Addressing these gaps in future research could provide a more nuanced understanding of the factors influencing neonatal outcomes in ART-conceived pregnancies.

## 5. Conclusions

In conclusion, our study provides valuable insights into the neonatal outcomes of IVF pregnancies using donor versus autologous material, highlighting key differences in perinatal health risks. The findings indicate that donor-conceived neonates, particularly those from multiple pregnancies, experience a higher prevalence of preterm birth, low birthweight, and increased rates of neonatal interventions, including ventilation and prolonged hospitalization. Additionally, our research underscores the association between donor conception and congenital malformations, particularly cardiac anomalies, suggesting potential underlying immunological and epigenetic mechanisms. Despite these concerns, the overall neonatal survival rates remained comparable between groups, reinforcing the efficacy of modern neonatal care in mitigating perinatal complications. By leveraging data from multiple tertiary centers and employing a comprehensive statistical approach, this study contributes to the growing body of literature on assisted reproductive technologies and neonatal health. However, continued research is necessary to further explore the biological mechanisms influencing these outcomes and to refine clinical protocols for optimizing neonatal well-being in IVF pregnancies. Future investigations should aim to incorporate larger cohorts, detailed maternal health parameters, and long-term follow-up studies to provide a more comprehensive understanding of the implications of gamete donation on child development.

## Figures and Tables

**Table 1:** Maternal baseline characteristics—singleton pregnancies after IVF donor vs. autologous material.

Singleton Pregnancies	IVF Donor Material (IVF-D)		IVF Autologous Material (IVF-A)		*p*-Value (IVF-D vs. IVF-A)	Unadjusted OR (95% CI)
	n	%	n	%		
Maternal age						
<35	5/670	13.5	282/670	44.5	***p* < 0.001** *	**1.63 (0.68–2.59)**
≥35	32/670	86.5	351/670	55.5		
Pregnancy-induced arterial hypertension	14/639	37.8	60/639	10	***p* < 0.001** *	**1.7 (0.98–2.42)**
Gestational diabetes	10/639	27	81/639	13.5	***p* = 0.027** *	**0.86 (0.1–1.63)**

**Table 2:** Maternal baseline characteristics—multiple pregnancies after IVF donor vs. autologous material.

Multiple Pregnancies	IVF Donor Material (IVF-D)		IVF Autologous Material (IVF-A)		*p*-Value (IVF-D vs. IVF-A)	Unadjusted OR (95% CI)
	n	%	n	%		
Maternal age						
<35	9/159	33.3	69/159	52.3	*p* = 0.056	0.78 (−0.08–1.65)
≥35	18/159	66.6	63/159	47.7		
Pregnancy-induced arterial hypertension	7/140	26	11/140	9.7	***p* = 0.032**	**1.17 (0.11–2.23)**
Gestational diabetes	2/140	7.4	14/140	12.4	*p* = 0.86	−0.57 (−2.11–0.97)

**Table 3:** Neonatal outcomes—singletons conceived through IVF donor vs. autologous material.

Singletons (n = 670)	IVF Donor Material (IVF-D) n = 37		IVF Autologous Material (IVF-A) n = 633			
	Median (IQR)	Range	Median (IQR)	Range		
Birthweight, grams	3130 (590)	920–4000	3190 (640)	590–4680		
Gestational age, weeks	38 (3)	28–40	38 (1)	25–42		
Apgar score at 1 min	9 (1)	3–10	9 (1)	1–10		
Apgar score at 5 min	7(1)	3–8	8 (1)	1–8		
Days of ventilation	0 (0)	0–10	0 (0)	0–56		
Days of hospitalization	3 (5)	3–49	3 (1)	2–100		
	**n**	**%**	**n**	**%**	***p*-value**	**Unadjusted OR** **(95% CI)**
Apgar 1′						
≤7	6/670	16.2	53/670	8.4	0.097	2.11 (0.84–5.3)
>7	31/670	83.8	580/670	91.6		
Apgar 5′						
≤7	5/670	13.5	25/670	4	**0.02**	**3.8** **(1.36–10.57)**
>7	32/670	86.5	608/670	96		
Asphyxia	6/670	16.2	53/670	8.4	0.097	2.11 (0.84–5.3)
Preterm birth (<37 weeks)	12/670	32.4	118/670	18.6	**0.038**	**2.09** **(1.02–4.29)**
Low birthweight (<2500 g)	8/670	21.6	72/670	11.4	0.062	2.14 (0.94–4.88)
Macrosomia (≥4000 g)	1/670	2.7	34/670	5.4	0.087	0.48 (0.06–3.67)
Ventilation required	6/670	16.2	27/670	4.3	**0.007**	**4.34** **(1.67–11.29)**
Days of ventilation						
>2	4/670	10.8	24/670	3.8	0.062	3.07 (1–9.37)
≤2	33/670	89.2	609/670	96.2		
Days of hospitalization						
>3	18/670	48.6	177/670	28	**0.008**	**2.44** **(1.25–4.75)**
≤3	19/670	51.4	456/670	72		
Congenital malformations	6/670	16.2	71/670	11.2	0.24	1.53 (0.61–3.8)
Neonatal death	1/670	2.7	3/670	0.5	0.2	5.83 (0.59–57.48)

**Table 4:** Neonatal outcomes—multiples conceived through IVF donor vs. autologous material.

Multiples (n = 318)	IVF Donor Material (IVF-D) n = 55		IVF Autologous Material (IVF-A) n = 263					
	Median (IQR)	Range	Median (IQR)	Range				
Birthweight, grams	2030 (540)	700–3800	2360 (1090)	600–4400				
Gestational age, weeks	34 (2)	27–39	36 (5)	25–39				
Apgar score at 1 min	7 (1.5)	4–10	8 (2)	1–10				
Apgar score at 5 min	6 (2)	4–8	7 (2)	1–8				
Days of ventilation	0 (0)	0–46	0 (0)	0–35				
Days of hospitalization	14 (24.5)	3–69	8 (26)	1–95				
	**n**	**%**	**n**	**%**		***p*-value**	**Unadjusted OR** **(95% CI)**	
Apgar 1′								
≤7	35/318	63.6	89/318	33.8		**<0.001**	**3.42** **(1.86–6.27)**	
>7	20/318	36.4	174/318	66.2				
Apgar 5′								
≤7	15/318	27.3	49/318	18.6		0.1	1.63 (0.83–3.2)	
>7	40/318	72.7	214/318	81.4				
Asphyxia	35/318	63.6	89/318	33.8	**<0.001**			**3.42** **(1.86–6.27)**
Preterm birth (<37 weeks)	50/318	90.9	179/318	68	**<0.001**			**4.69** **(1.8–12.19)**
Low birthweight (<2500 g)	47/318	85.5	150/318	57	**<0.001**			**4.42** **(2.01–9.73)**
Macrosomia (≥4000 g)	0/318	0	4/318	1.5	1			0.52 (0.02–9.78)
Ventilation required	13/318	23.6	64/318	24.3	0.6			0.96 (0.48–1.9)
Days of ventilation								
>2	13/318	23.6	41/318	15.6	0.1			1.67 (0.82–3.39)
≤2	42/318	76.4	222/318	84.4				
Days of hospitalization								
>3	53/318	96.4	189/318	71.9	**<0.001** *			**10.37** **(2.46–43.66)**
≤3	2/318	3.6	74/318	28.1				
Congenital malformations	15/318	27.3	41/318	15.6	**0.034** *			**2.03** **(1.02–4.01)**
Neonatal death	1/318	1.8	5/318	1.9	0.68			0.95 (0.1–8.34)

## Data Availability

Data available upon request.

## Supplementary Materials

The following supporting information can be downloaded at https://www.mdpi.com/article/10.3390/life15040578/s1, Table S1. Congenital malformations (detailed); Table S2. Days of ventilation and hospital stay; Table S3. Kendall’s Tau correlations; Table S4. Mann-Whitney U test assumption checks; Table S5. Male vs female as a covariate; Table S6. Weight percentiles-statistical analysis.
