# Supplementary material for: Neonatal Health Following IVF: Own Versus Donor Material in Singleton and Multiple Pregnancies
Source: Life (Basel). 2025 Apr 1;15(4):578. doi: 10.3390/life15040578 (PMC12029059; doi:10.3390/life15040578)
Supplement: Supplementary file 1 [file life-15-00578-s001.zip › Table S1. Congenital malformations (detailed).pdf]

SINGLETONS (670)		
Types of congenital malformations	Number of children with congenital malformations from ART using own material 71/633 (11.2%)	Number of children with congenital malformations from ART using donor material 6/37 (16.2%)
**Cardiac**	**24 (3.8%)**	**5 (13.5%)**
Patent foramen ovale	11	2
Patent ductus arteriosus	2	
Atrial septal aneurysm		1
Atrial septal defect	4	
Ventricular septal defect	4	1
Coarctation of the aorta	1	
Pulmonary artery stenosis	2	
Congenital tricuspid valve dysplasia		1
**Respiratory**	**0**	**0**
**Gastro-intestinal**	**9 (1.4%)**	**0**
Ankyloglossia	8	
Cleft lip/palate	1	
**Genito-urinary**	**20 (3.1%)**	**1 (2.7%)**
Hydronephrosis/Urinary tract dilation	7	1
Unilateral kidney agenesis	1	
Ovarian cyst	1	
Hypospadias	4	
Cryptorchidism	3	
Inguinal hernia	2	
Hydrocele	2	
**Limbs**	**4 (0.6%)**	**0**
Syndactyly	2	
Metatarsus adductus/Clubfoot	2	
**Chromosomal/Genetic**	**3 (0.5%)**	**0**
Sex chromosome mosaicism	1	
GJB2 gene mutation	1	
Hydrops fetalis	1	
**Dermatological and Soft Tissue**	**11 (1.7%)**	**0**
Preauricular cyst/pit	2	
Craniotabes	3	
Cavernous hemangioma	1	
Coccygeal pit/Sacral dimple	2	
Congenital dermal melanocytosis	3	
**TWINS, TRIPLETS (318)**		

Types of congenital malformations	Number of children with congenital malformations from ART using own material<br>41/263 (15.6%)	Number of children with congenital malformations from ART using donor material<br>15/55 (27.3%)
**Cardiac**	**16 (6%)**	**10 (18.2%)**
Patent foramen ovale	5	4
Patent ductus arteriosus	1	
Atrial septal aneurysm	1	
Atrial septal defect	1	2
Ventricular septal defect	3	1
Coarctation of the aorta		2
Pulmonary artery stenosis	3	
Hypertrophic cardiomyopathy	1	
Persistent left superior vena cava	1	
Congenital tricuspid valve dysplasia		1
**Respiratory**	**1 (0.4%)**	**0**
Diaphragmatic hernia	1	
**Gastro-intestinal**	**3 (1.1%)**	**0**
Ankyloglossia	3	
**Genito-urinary**	**13 (4.9%)**	**3 (5.4%)**
Hydronephrosis/Urinary tract dilation	6	1
Horseshoe kidney		1
Unilateral kidney agenesis	1	
Hypospadias	1	
Cryptorchidism	2	
Inguinal hernia		1
Hydrocele	3	
**Limbs**	**2 (0.8%)**	**0**
Polydactyly hand or foot	1	
Metatarsus adductus/Clubfoot	1	
**Chromosomal/Genetic**	**1 (0.4%)**	**1 (1.8%)**
Trisomy 18	1	
Coffin-Siris syndrome		1
**Dermatological and Soft Tissue**	**5 (1.9%)**	**1 (1.8%)**
Branchial cleft cyst	1	
Preauricular cyst/pit		1
Cavernous hemangioma	1	
Lymphatic malformation	1	
Unspecified multiple malformations	2	
