# Supplementary material for: Neonatal Health Following IVF: Own Versus Donor Material in Singleton and Multiple Pregnancies
Source: Life (Basel). 2025 Apr 1;15(4):578. doi: 10.3390/life15040578 (PMC12029059; doi:10.3390/life15040578)

SINGLETONS

Table S2a. Mann-Whitney U test evaluating singletons conceived through IVF Donor vs. Autologous material and days of ventilation/hospital stay.

	U	p
Days of invasive or non-invasive ventilation	13082.000	< 0.001
Days of hospitalisation	14783.000	< 0.001

Table S2b. Mann-Whitney U test evaluating singletons – group descriptives.

Group	N	Mean	SD	SE	Coefficient of variation	Mean Rank	Sum Rank
**Days of ventilation**							
Donor	37	0.784	2.287	0.376	2.918	372.568	13785.000
Own	633	0.423	3.069	0.122	7.249	333.333	211000.000
**Length of hospital stay**							
Donor	37	8.676	11.865	1.951	1.368	418.541	15486.000
Own	633	5.641	9.979	0.397	1.769	330.646	209299.000

Bar Plots

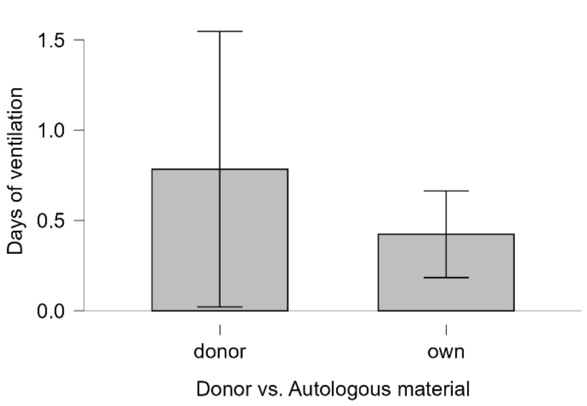

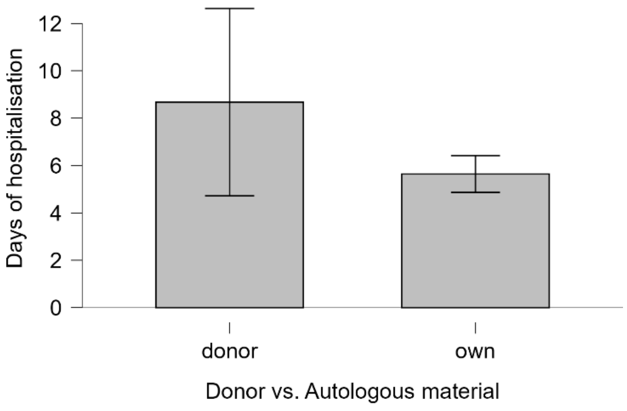

MULTIPLES

Table S2c. Mann-Whitney U test evaluating multiples conceived through IVF Donor vs. Autologous material and days of ventilation/hospital stay.

	U	p
Days of invasive or non-invasive ventilation	7269.500	0.469
Days of hospitalisation	9468.000	< 0.001

Table S2d. Mann-Whitney U test evaluating multiples – group descriptives.

Group	N	Mean	SD	SE	Coefficient of variation	Mean Rank	Sum Rank
**Days of ventilation**							
Donor	55	2.436	7.018	0.946	2.880	160.173	8809.500
Own	263	1.962	5.338	0.329	2.721	159.359	41911.500
**Length of hospital stay**							
Donor	55	21.418	16.120	2.174	0.753	200.145	11008.000
Own	263	17.152	19.787	1.220	1.154	151.000	39713.000

Raincloud Plots

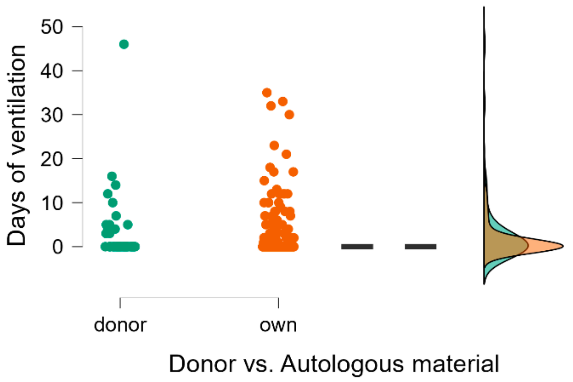

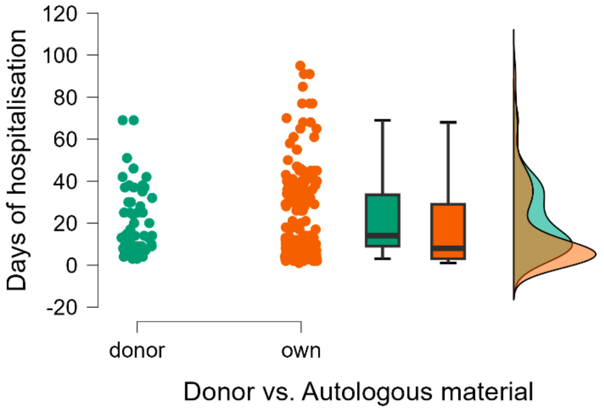

Supplement: Supplementary file 1 [file life-15-00578-s001.zip › Table S2. Days of ventilation and hospital stay.pdf]
