# Supplementary material for: Neonatal Health Following IVF: Own Versus Donor Material in Singleton and Multiple Pregnancies
Source: Life (Basel). 2025 Apr 1;15(4):578. doi: 10.3390/life15040578 (PMC12029059; doi:10.3390/life15040578)

SINGLETONS

Kendall's Tau Correlations

Variable		Gestational age (weeks)	Birthweight (grams)	Apgar score 1'	Apgar score 5'	Days of ventilation	Days of hospitalisation	Age at birth
1. Gestational age (weeks)	Kendall's Tau B	—						
	p-value	—						
2. Birthweight (grams)	Kendall's Tau B	0.403	—					
	p-value	< .001	—					
3. Apgar score 1'	Kendall's Tau B	0.250	0.280	—				
	p-value	< .001	< .001	—				
4. Apgar score 5'	Kendall's Tau B	0.288	0.306	0.693	—			
	p-value	< .001	< .001	< .001	—			
5. Days of ventilation	Kendall's Tau B	-0.308	-0.246	-0.346	-0.390	—		
	p-value	< .001	< .001	< .001	< .001	—		
6. Days of hospitalisation	Kendall's Tau B	-0.351	-0.331	-0.347	-0.354	0.385	—	
	p-value	< .001	< .001	< .001	< .001	< .001	—	
7. Age at birth	Kendall's Tau B	-0.078	-0.074	-0.094	-0.097	0.073	0.050	—
	p-value	0.007	0.006	0.002	0.002	0.023	0.094	—

Assumption checks

Shapiro-Wilk Test for Multivariate Normality

Shapiro-Wilk	p
0.808	< .001

Shapiro-Wilk Test for Bivariate Normality

			Shapiro-Wilk	p
Gestational age (weeks)	-	Birthweight (grams)	0.953	< .001
Gestational age (weeks)	-	Apgar score 1'	0.819	< .001
Gestational age (weeks)	-	Apgar score 5'	0.769	< .001
Gestational age (weeks)	-	Days of ventilation	0.516	< .001
Gestational age (weeks)	-	Days of hospitalisation	0.616	< .001
Gestational age (weeks)	-	Age at birth	0.882	< .001
Birthweight (grams)	-	Apgar score 1'	0.885	< .001
Birthweight (grams)	-	Apgar score 5'	0.857	< .001
Birthweight (grams)	-	Days of ventilation	0.643	< .001
Birthweight (grams)	-	Days of hospitalisation	0.806	< .001
Birthweight (grams)	-	Age at birth	0.962	< .001
Apgar score 1'	-	Apgar score 5'	0.786	< .001
Apgar score 1'	-	Days of ventilation	0.448	< .001
Apgar score 1'	-	Days of hospitalisation	0.544	< .001
Apgar score 1'	-	Age at birth	0.834	< .001
Apgar score 5'	-	Days of ventilation	0.372	< .001
Apgar score 5'	-	Days of hospitalisation	0.473	< .001
Apgar score 5'	-	Age at birth	0.785	< .001
Days of ventilation	-	Days of hospitalisation	0.297	< .001
Days of ventilation	-	Age at birth	0.566	< .001
Days of hospitalisation	-	Age at birth	0.658	< .001

Correlation plot

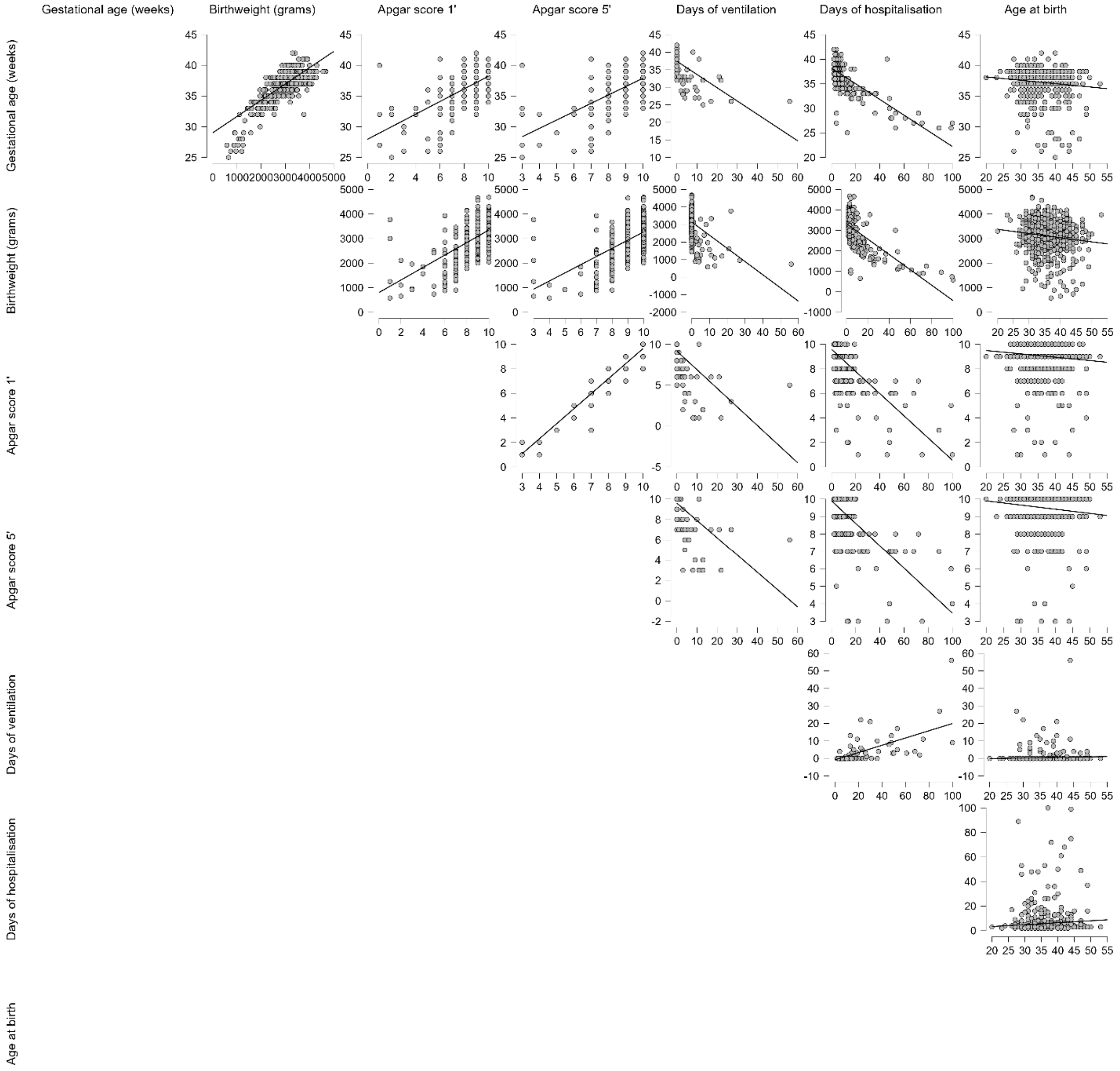

Kendall's tau B heatmap

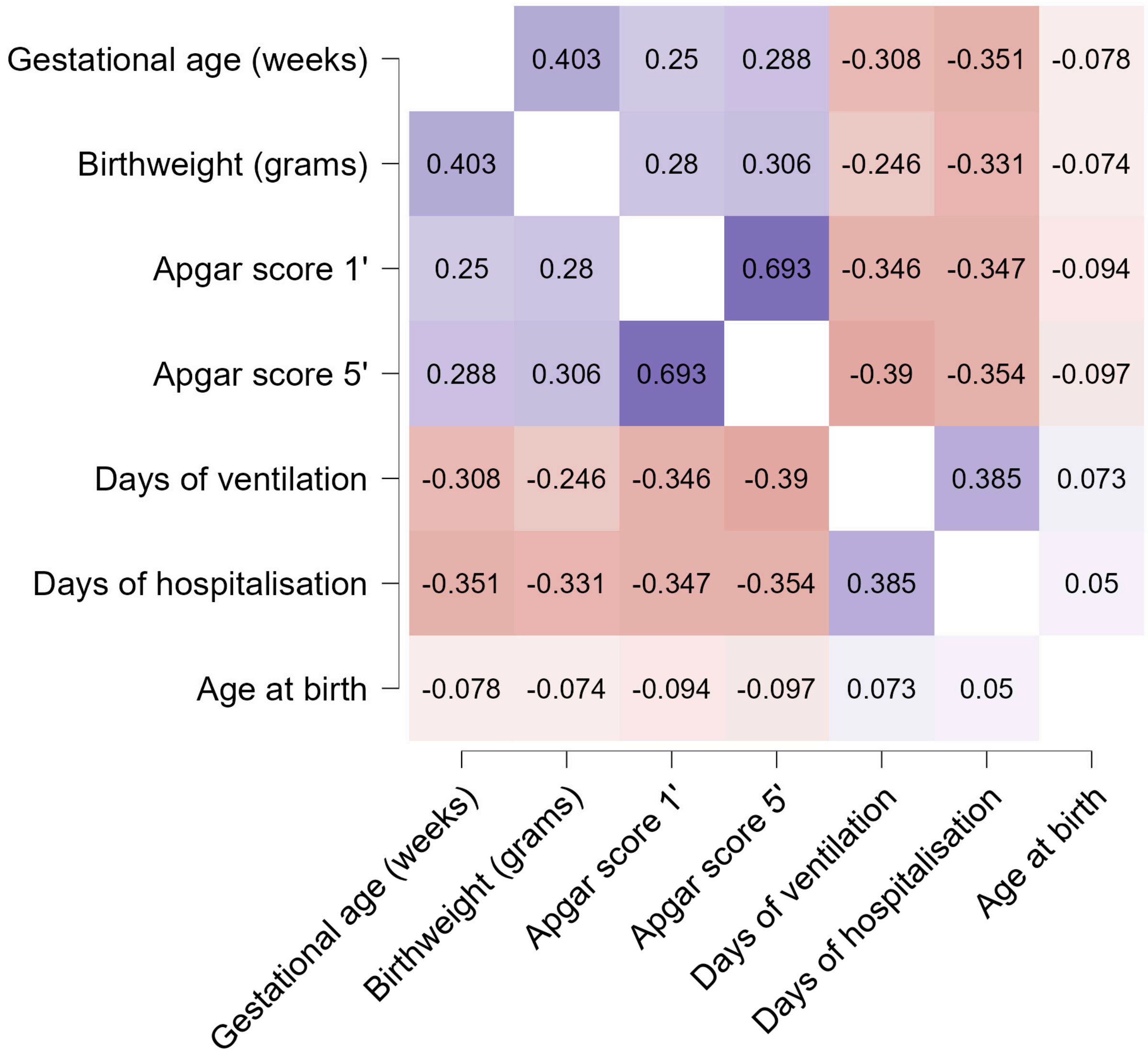
Gestational age (weeks)
Birthweight (grams)
Apgar score 1'
Apgar score 5'
Days of ventilation
Days of hospitalisation
Age at birth
0.403 0.25 0.288 -0.308 -0.351 -0.078
0.403 0.28 0.306 -0.246 -0.331 -0.074
0.25 0.28 0.693 -0.346 -0.347 -0.094
0.288 0.306 0.693 -0.39 -0.354 -0.097
-0.308 -0.246 -0.346 -0.39 0.385 0.073
-0.351 -0.331 -0.347 -0.354 0.385 0.05
-0.078 -0.074 -0.094 -0.097 0.073 0.05
Gestational age (weeks)
Birthweight (grams)
Apgar score 1'
Apgar score 5'
Days of ventilation
Days of hospitalisation
Age at birth

MULTIPLES

Kendall's Tau Correlations

Variable		Gestational age (weeks)	Birthweight (grams)	Apgar score 1'	Apgar score 5'	Days of ventilation	Days of hospitalisation	Age at birth
1. Gestational age (weeks)	Kendall's Tau B	—						
	p-value	—						
2. Birthweight (grams)	Kendall's Tau B	0.727	—					
	p-value	< .001	—					
3. Apgar score 1'	Kendall's Tau B	0.620	0.616	—				
	p-value	< .001	< .001	—				
4. Apgar score 5'	Kendall's Tau B	0.588	0.569	0.875	—			
	p-value	< .001	< .001	< .001	—			
5. Days of ventilation	Kendall's Tau B	-0.548	-0.483	-0.494	-0.498	—		
	p-value	< .001	< .001	< .001	< .001	—		
6. Days of hospitalisation	Kendall's Tau B	-0.701	-0.672	-0.593	-0.541	0.488	—	
	p-value	< .001	< .001	< .001	< .001	< .001	—	
7. Age at birth	Kendall's Tau B	-0.002	-0.061	-0.117	-0.094	-0.053	0.050	—
	p-value	0.964	0.115	0.005	0.028	0.231	0.204	—

Assumption checks

Shapiro-Wilk Test for Multivariate Normality

Shapiro-Wilk	p
0.923	< .001

Shapiro-Wilk Test for Bivariate Normality

			Shapiro-Wilk	p
Gestational age (weeks)	-	Birthweight (grams)	0.995	0.356
Gestational age (weeks)	-	Apgar score 1'	0.951	< .001
Gestational age (weeks)	-	Apgar score 5'	0.954	< .001
Gestational age (weeks)	-	Days of ventilation	0.727	< .001
Gestational age (weeks)	-	Days of hospitalisation	0.842	< .001
Gestational age (weeks)	-	Age at birth	0.954	< .001
Birthweight (grams)	-	Apgar score 1'	0.983	< .001
Birthweight (grams)	-	Apgar score 5'	0.988	< .001
Birthweight (grams)	-	Days of ventilation	0.842	< .001
Birthweight (grams)	-	Days of hospitalisation	0.961	< .001
Birthweight (grams)	-	Age at birth	0.985	< .001
Apgar score 1'	-	Apgar score 5'	0.944	< .001
Apgar score 1'	-	Days of ventilation	0.686	< .001
Apgar score 1'	-	Days of hospitalisation	0.833	< .001
Apgar score 1'	-	Age at birth	0.953	< .001
Apgar score 5'	-	Days of ventilation	0.660	< .001
Apgar score 5'	-	Days of hospitalisation	0.825	< .001
Apgar score 5'	-	Age at birth	0.934	< .001
Days of ventilation	-	Days of hospitalisation	0.671	< .001
Days of ventilation	-	Age at birth	0.696	< .001
Days of hospitalisation	-	Age at birth	0.876	< .001

Correlation plot

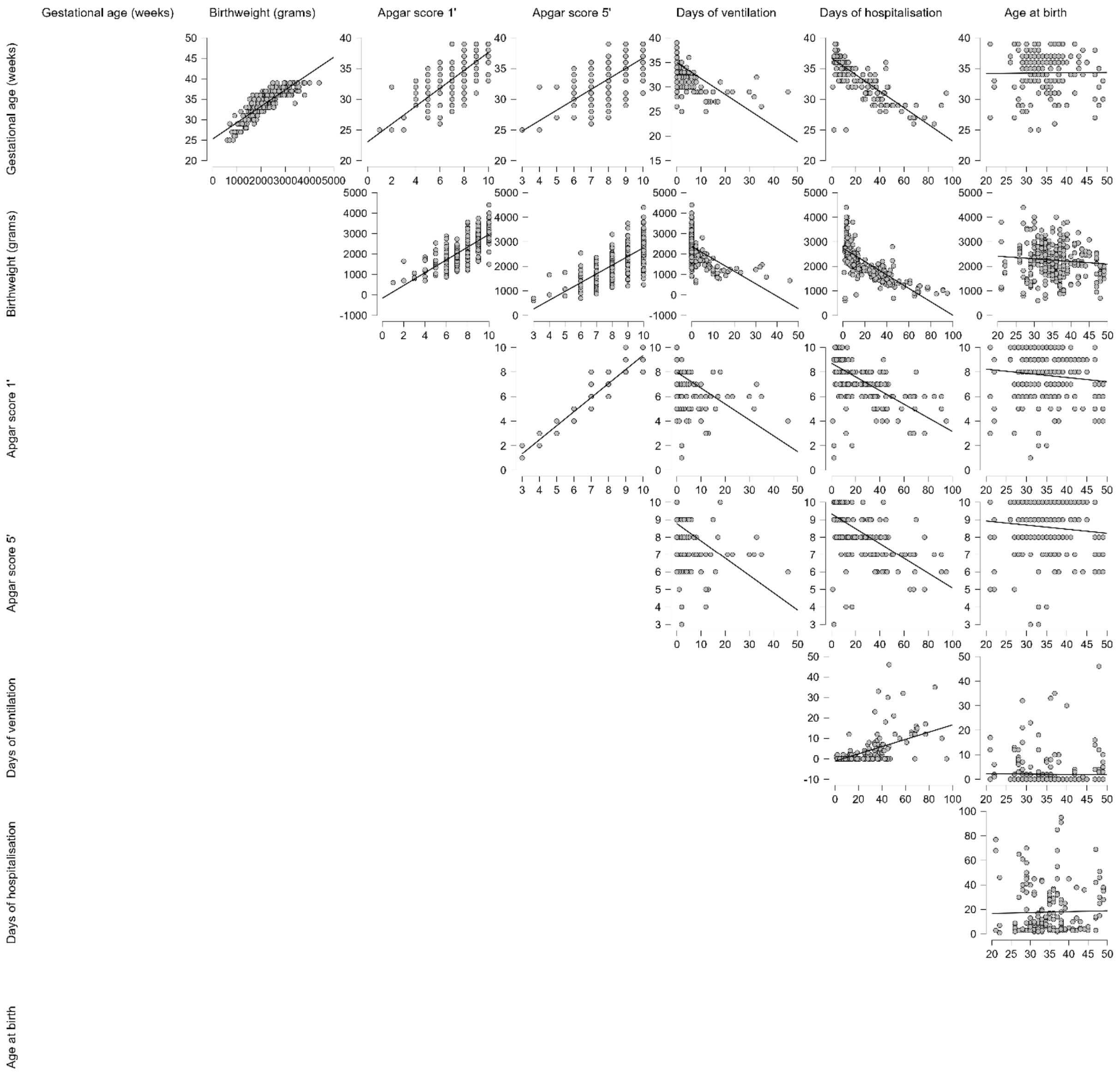

Kendall's tau B heatmap

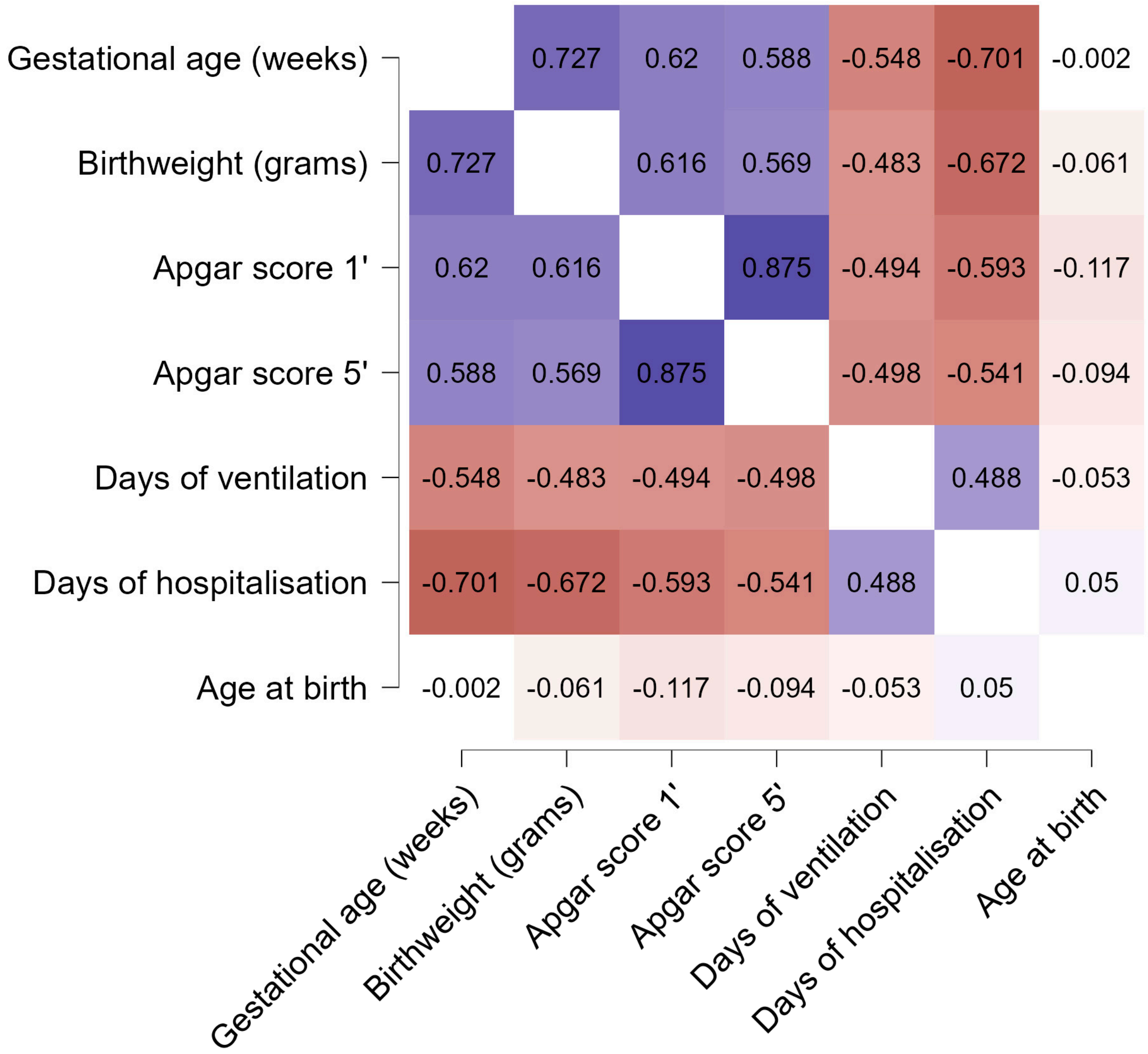

Supplement: Supplementary file 1 [file life-15-00578-s001.zip › Table S3. Kendall's Tau correlations.pdf]
