# Supplementary material for: Neonatal Health Following IVF: Own Versus Donor Material in Singleton and Multiple Pregnancies
Source: Life (Basel). 2025 Apr 1;15(4):578. doi: 10.3390/life15040578 (PMC12029059; doi:10.3390/life15040578)
Supplement: Supplementary file 1 [file life-15-00578-s001.zip › Table S4. Mann-Whitney U test assumption checks.pdf]

SINGLETONS

Test of Normality (Shapiro-Wilk)

Residuals	W	p
Days of invasive or non-invasive ventilation	0.139	< .001
Days of hospitalisation	0.337	< .001

Note. Significant results suggest a deviation from normality.

Test of Equality of Variances (Brown-Forsythe)

	F	df_1	df_2	p
Days of invasive or non-invasive ventilation	0.494	1	668	0.482
Days of hospitalisation	2.893	1	668	0.089

MULTIPLES

Test of Normality (Shapiro-Wilk)

Residuals	W	p
Days of invasive or non-invasive ventilation	0.435	< .001
Days of hospitalisation	0.776	< .001

Note. Significant results suggest a deviation from normality.

Test of Equality of Variances (Brown-Forsythe)

	F	df_1	df_2	p
Days of invasive or non-invasive ventilation	0.319	1	316	0.572
Days of hospitalisation	0.049	1	316	0.825
