# Supplementary material for: Neonatal Health Following IVF: Own Versus Donor Material in Singleton and Multiple Pregnancies
Source: Life (Basel). 2025 Apr 1;15(4):578. doi: 10.3390/life15040578 (PMC12029059; doi:10.3390/life15040578)
Supplement: Supplementary file 1 [file life-15-00578-s001.zip › Table S5. Male vs female as a covariate.pdf]

Evaluating sex as a covariate for autologous versus donor IVF neonatal outcomes

SINGLETONS

Descriptive Statistics

		Valid	Median	Std. Deviation	Minimum	Maximum
Gestational.age (weeks)	female	329	38.000	2.293	25.000	42.000
Gestational.age (weeks)	male	341	38.000	2.103	26.000	41.000
Birthweight (grams)	female	329	3150.000	637.504	580.000	4680.000
Birthweight (grams)	male	341	3200.000	590.716	740.000	4300.000
Apgar score 1 minute	female	329	9.000	1.492	1.000	10.000
Apgar score 1 minute	male	341	9.000	1.202	1.000	10.000
Days of ventilation	female	329	0.000	2.370	0.000	27.000
Days of ventilation	male	341	0.000	3.558	0.000	56.000
Days.of.hospitalisation	female	329	3.000	11.156	2.000	100.000
Days.of.hospitalisation	male	341	3.000	8.989	2.000	99.000

Boxplots

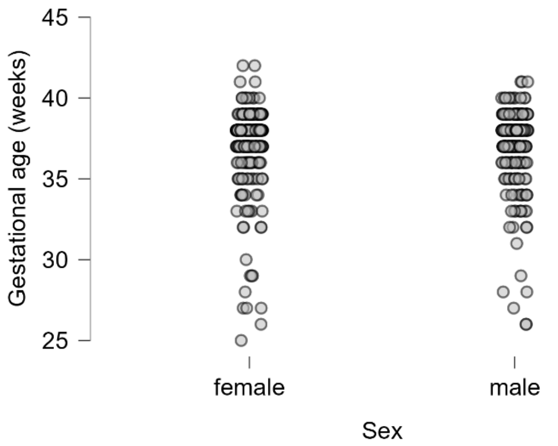

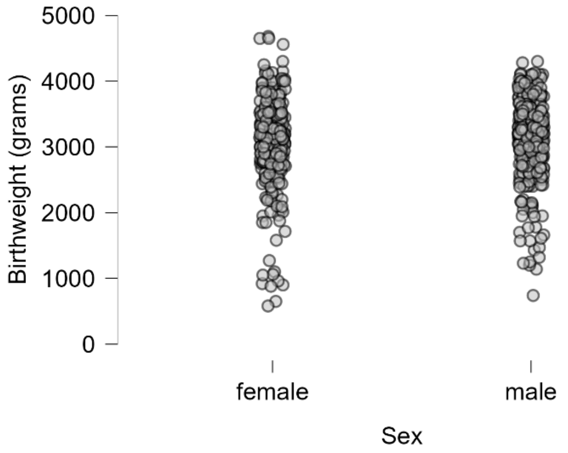

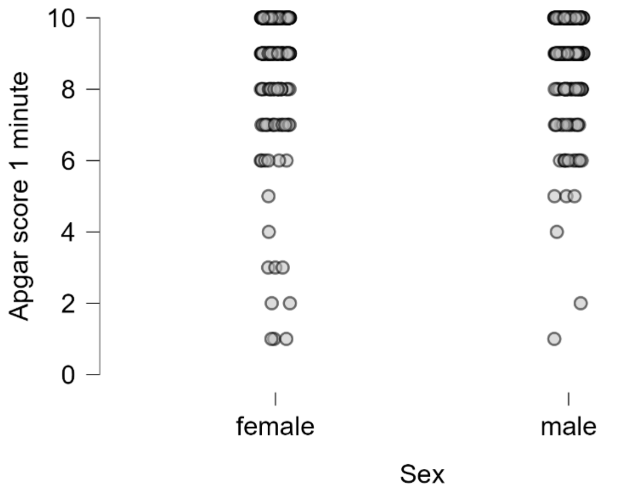
Apgar score 1 minute
0
2
4
6
8
10
female
male
Sex

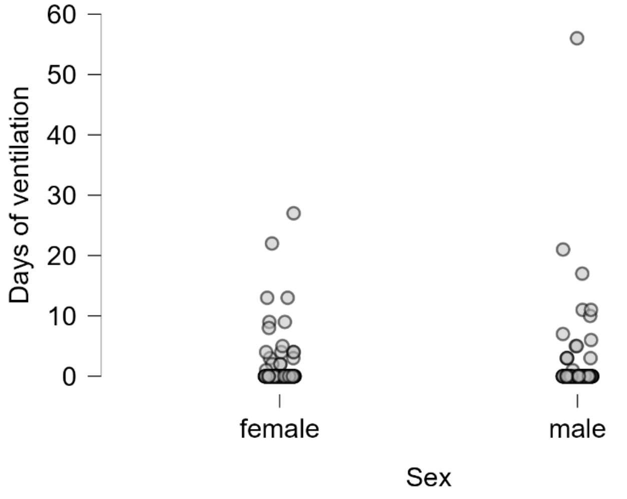
Days of ventilation
0
10
20
30
40
50
60
female
male
Sex

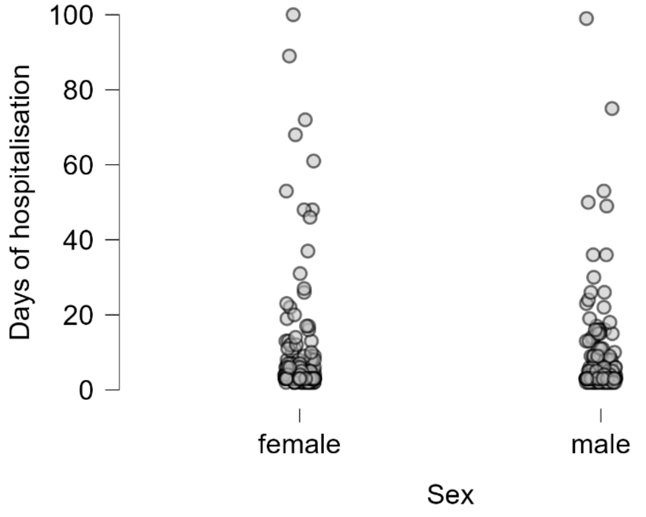
Days of hospitalisation
0
20
40
60
80
100
female
male
Sex

Independent Samples T-Test

	U	df	p
Gestational.age..weeks.	56220.000		0.959
Birthweight..grams.	54817.000		0.610
Apgar.score.1.	59801.500		0.109
Days.of.invasive.or.non.invasive.ventilation	56675.500		0.537
Days.of.hospitalisation	55389.000		0.743

Note. Mann-Whitney U test.

Test of Normality (Shapiro-Wilk)

Residuals	W	p
Gestational.age..weeks.	0.759	< .001
Birthweight..grams.	0.938	< .001
Apgar.score.1.	0.649	< .001
Days.of.invasive.or.non.invasive.ventilation	0.138	< .001
Days.of.hospitalisation	0.331	< .001

Note. Significant results suggest a deviation from normality.

Test of Equality of Variances (Brown-Forsythe)

	F	df_1	df_2	p
Gestational.age..weeks.	0.046	1	668	0.830
Birthweight..grams.	0.143	1	668	0.705
Apgar.score.1.	2.935	1	668	0.087
Days.of.invasive.or.non.invasive.ventilation	0.076	1	668	0.782
Days.of.hospitalisation	0.262	1	668	0.609

MULTIPLES

Descriptive Statistics

		Valid	Median	Std. Deviation	Minimum	Maximum
Gestational.age (weeks)	Female	161	35.000	3.161	25.000	39.000
Gestational.age (weeks)	Male	157	35.000	3.322	25.000	39.000
Birthweight (grams)	Female	161	2200.000	682.586	600.000	4400.000
Birthweight (grams)	Male	157	2300.000	726.525	790.000	4000.000
Apgar score 1 minute	Female	161	8.000	1.659	1.000	10.000
Apgar score 1 minute	Male	157	8.000	1.694	2.000	10.000
Days of ventilation	Female	161	0.000	6.042	0.000	46.000
Days of ventilation	Male	157	0.000	5.243	0.000	33.000
Days.of.hospitalisation	Female	161	9.000	19.215	1.000	95.000
Days.of.hospitalisation	Male	157	9.000	19.356	2.000	91.000

Boxplots

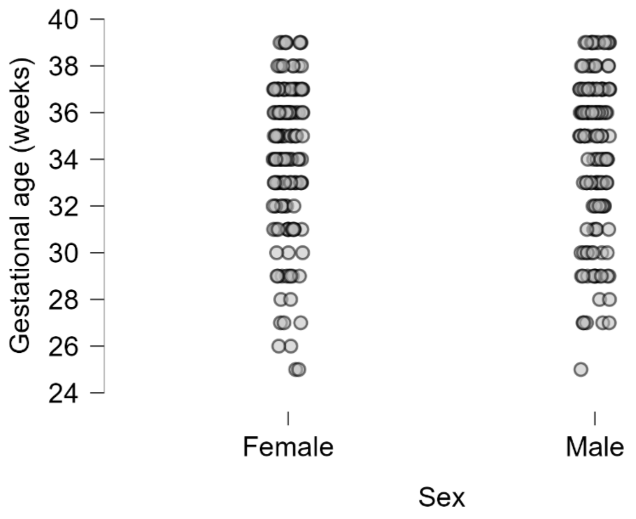

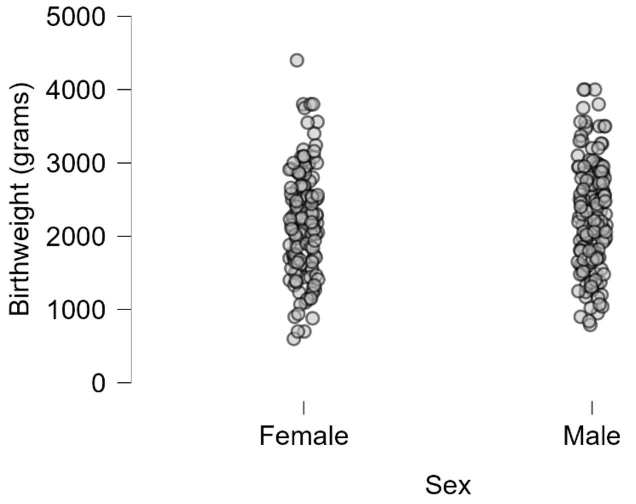

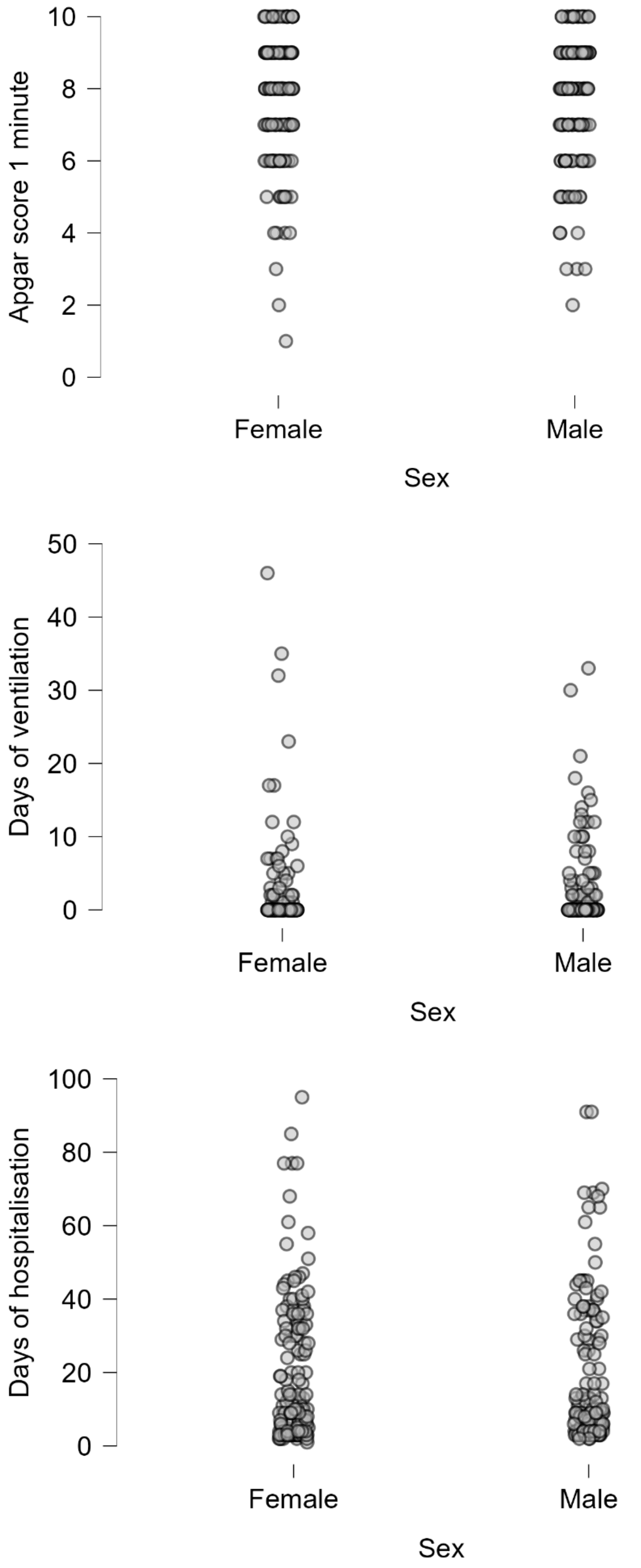

Apgar score 1 minute
0
2
4
6
8
10
Female
Male
Sex
Days of ventilation
0
10
20
30
40
50
Female
Male
Sex
Days of hospitalisation
0
20
40
60
80
100
Female
Male
Sex

Independent Samples T-Test

	U	df	p
Birthweight..grams.	11703.500		0.254
Gestational.age..weeks.	11992.500		0.428
Apgar.score.1.	12218.500		0.602
Days.of.invasive.or.non.invasive.ventilation	11960.500		0.271
Days.of.hospitalisation	12761.500		0.881

Note. Mann-Whitney U test.

Test of Normality (Shapiro-Wilk)

Residuals	W	p
Birthweight..grams.	0.994	0.296
Gestational.age..weeks.	0.940	< .001
Apgar.score.1.	0.918	< .001
Days.of.invasive.or.non.invasive.ventilation	0.431	< .001
Days.of.hospitalisation	0.783	< .001

Note. Significant results suggest a deviation from normality.

Test of Equality of Variances (Brown-Forsythe)

	F	df_1	df_2	p
Birthweight..grams.	2.073	1	316	0.151
Gestational.age..weeks.	0.589	1	316	0.443
Apgar.score.1.	0.122	1	316	0.727
Days.of.invasive.or.non.invasive.ventilation	0.175	1	316	0.676
Days.of.hospitalisation	0.132	1	316	0.717
