# Supplementary material for: Neonatal Health Following IVF: Own Versus Donor Material in Singleton and Multiple Pregnancies
Source: Life (Basel). 2025 Apr 1;15(4):578. doi: 10.3390/life15040578 (PMC12029059; doi:10.3390/life15040578)
Supplement: Supplementary file 1 [file life-15-00578-s001.zip › Table S6. Weight percentiles - statistical analysis.pdf]

SINGLETONS

Own.Donor.material	SGA/notSGA		
	SGA	not SGA	Total
donor	1	36	37
own	21	612	633
Total	22	648	670

Note. Each cell displays the observed counts

Chi-Squared Tests

	Value	df	p
X^2	0.042	1	0.838
N	670		

Odds Ratio

	Odds Ratio	95% Confidence Intervals		p
		Lower	Upper	
Odds ratio	0.810	0.106	6.189	
Fisher's exact test	0.810	0.039	∞	0.719

Note. For all tests, the alternative hypothesis specifies that group *donor* is greater than *own* .

MULTIPLES

Own.Donor.material	SGA/not SGA		
	SGA	not SGA	Total
donor	8	47	55
own	23	240	263
Total	31	287	318

Note. Each cell displays the observed counts

Chi-Squared Tests

	Value	df	p
X^2	1.739	1	0.187
N	318		

Odds Ratio

	Odds Ratio	95% Confidence Intervals		p
		Lower	Upper	
Odds ratio	1.776	0.749	4.210	
Fisher's exact test	1.772	0.756	∞	0.143

Note. For all tests, the alternative hypothesis specifies that group *donor* is greater than *own* .
